# Face Recognition Increases during Saccade Preparation

**DOI:** 10.1371/journal.pone.0093112

**Published:** 2014-03-26

**Authors:** Hai Lin, Joshua D. Rizak, Yuan-ye Ma, Shang-chuan Yang, Lin Chen, Xin-tian Hu

**Affiliations:** 1 Key Laboratory of Animal Models and Human Disease Mechanisms, Kunming Institute of Zoology, Chinese Academy of Sciences, Kunming, Yunnan, China; 2 Key Laboratory of Animal Models and Human Disease Mechanisms, Kunming Institute of Zoology, Chinese Academy of Sciences, Kunming, Yunnan, China; 3 State Key Laboratory of Brain and Cognitive Science, Institute of Biophysics, Chinese Academy of Sciences, Beijing, China; 4 University of Chinese Academy of Sciences, Beijing, China; 5 State Key Laboratory of Brain and Cognitive Science, Institute of Biophysics, Chinese Academy of Sciences, Beijing, China; University of Leicester, United Kingdom

## Abstract

Face perception is integral to human perception system as it underlies social interactions. Saccadic eye movements are frequently made to bring interesting visual information, such as faces, onto the fovea for detailed processing. Just before eye movement onset, the processing of some basic features, such as the orientation, of an object improves at the saccade landing point. Interestingly, there is also evidence that indicates faces are processed in early visual processing stages similar to basic features. However, it is not known whether this early enhancement of processing includes face recognition. In this study, three experiments were performed to map the timing of face presentation to the beginning of the eye movement in order to evaluate pre-saccadic face recognition. Faces were found to be similarly processed as simple objects immediately prior to saccadic movements. Starting ∼ 120 ms before a saccade to a target face, independent of whether or not the face was surrounded by other faces, the face recognition gradually improved and the critical spacing of the crowding decreased as saccade onset was approaching. These results suggest that an upcoming saccade prepares the visual system for new information about faces at the saccade landing site and may reduce the background in a crowd to target the intended face. This indicates an important role of pre-saccadic eye movement signals in human face recognition.

## Introduction

Face recognition is an integral component of human perception as it mediates social interactions. As such, there have been a substantial number of studies evaluating face perception. Face recognition had been shown to be selectively impaired in comparison to equally challenging object recognition [1]. Ultimately, this led to the realization that different brain regions were used in the recognition of faces and objects [2]. A specific area in the human fusiform gyrus, the fusiform face area (FFA), has been identified using FMRI to be specialized for face processing [3], [4]. In addition, specific populations of cells located in the temporal cortex have been found to respond selectively to faces using single electrode recordings in monkeys [5], identifying these cells as key players in the processing of faces.

Moreover, faces are better recognized when they are presented in the correct orientation rather than upside-down [6]–[8] and facial features are better identified in the context of a complete face rather than as part of a scrambled face or as an individual feature [9], [10]. This suggested that humans might process faces in a holistic manner [11].

However, when an interesting object, such as a face, first enters the peripheral visual field it attracts attention and is usually responded to by a rapid eye movement. This movement is known as a saccade and it acts to bring the object into the high-acuity foveal vision for further processing. Interestingly, the increased processing of the saccade targeted object begins before the eye movement [12], [13]. This has been shown through the improving performance of observers in visual orientation-discrimination tasks as saccade onset approaches. This type of increased pre-saccadic visual processing occurs ∼ 100 ms before the saccade and has been compared to an adjustment in the physical contrast between visual stimuli [14]–[17]. These earlier studies demonstrated that an upcoming saccade can increase the recognition of basic features, such as the color or orientation, of objects in the periphery [17], [18]. Interestingly, there is some evidence that indicates faces are also processed in early visual processing stages, similar to basic features [19]. However, it is not known whether this increased processing includes face recognition.

**Figure 1:**
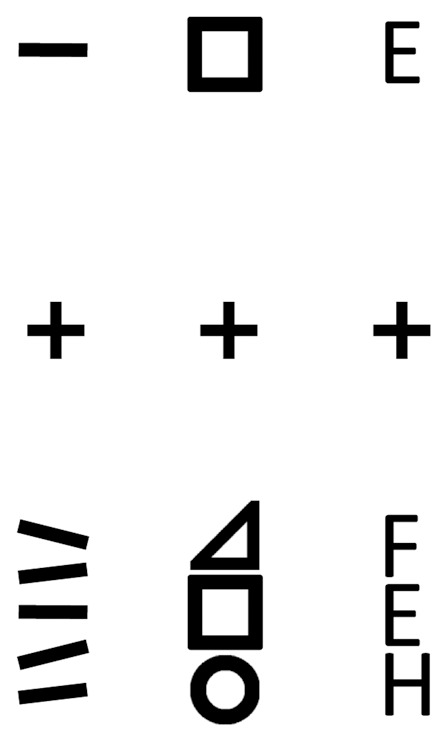
The crowding effects of neighboring elements. While fixating on the crosses, it is easy to identify the line orientation, shape or letter on the top half of the panel, while difficult to identify the middle line orientation, shape or letter on the bottom half.

Furthermore, in the natural visual world, objects, including faces, rarely exist on their own but rather in clusters. Unfortunately, simple objects, let alone faces, in the peripheral visual field that would be easily identifiable become challenging to recognize when they are presented in close proximity to other similar objects. This occurrence is called crowding [20], [21]. This effect can be experienced by fixating on the crosses in Figure 1. In doing so, the identification of the orientation, shape or letter of the middle line in the bottom half of the panel becomes difficult or impossible, where as items in the upper panel can be identified in the peripheral view. This effect of crowding has been demonstrated using letters, digits, gratings, and faces [22]–[25]. This suggests that the perception hierarchy in the visual system is affected at multiple stages by crowding [26]. The crowding effect is related to the target-distractor distance. When this distance increases to a specific point, these crowding effects become null. This is known as the “critical spacing”. Bouma’s law describing the relationship between objects in a crowd to their spatial distances states that the “critical spacing” is roughly half of the eccentricity of the target [20], [27], which suggests that the distances between faces in a crowd play a role in the ability to accurately recognize a face.

This improved pre-saccadic processing has also been recently linked to the identification of objects in a crowd. A study by Harrison et al. [18] found that the identification of a non-facial object surrounded by competing objects increased dramatically ∼ 50ms prior to a saccade in an object orientation-discrimination task. It is notable that these changes in perception occurred without a positional change of the retina and they were related to a 0.5 fold change in the “critical spacing” between the target and distractor stimuli [18]. This suggested that pre-saccade perception might aid in identifying objects, including faces, in a natural/social setting.

In this study, the relationship between pre-saccade perception and face recognition was evaluated using similar paradigms designed previously [17], [18], in order to determine whether there is a similar eye movement preparation effect on the processing of faces in either crowded or isolated states and whether this processing aids face recognition.

## Materials and Methods

### Ethics statement

This study was conducted according to the principles expressed in the Declaration of Helsinki and had approval from the Human Research Ethics Committee of the Institute of Biophysics, Chinese Academy of Sciences. All observers provided written informed consent for the collection of data and subsequent analysis.

### Participants

Five Chinese observers, including the first author (age 23–26 years, two males/three females, right-eye dominant) were tested in all experiments. All observers, with exception of the author, were naive regarding the purpose of the study. However, two participants had been psychophysically trained. The observers had normal or corrected-to normal vision.

### Apparatus and stimuli

Observers sat in a silent and dimly lit room, head positioned on a chin rest. Stimuli were presented at a 61 cm distance on a 17-inch screen (1280×1024 pixels, 60 Hz vertical refresh). An EyeLink 1000 Desktop Mount (SR Research, Ottawa, Canada) recorded the right eye’s gaze position at 1000 Hz. A computer running MATLAB (MathWorks, Natick, USA) with standard toolboxes [28]–[30] controlled the stimulus presentation and response collection.

The stimuli included 20 faces with neutral expressions, half of which were male. The images were obtained from the Matsumoto and Ekman’s Japanese and Caucasian Neutral Faces (JACNeuF) database (University of California-San Francisco (UCSF), San Francisco, USA) with their expressed permission and consent. Using MATLAB (MathWorks, Natick, USA), all stimuli were grayscale filtered and Gaussian band-pass filtered for spatial frequency with a center spatial frequency of 0.5 cycles/pixel and a Gaussian function sigma value of 0.2 cycles/pixel. All images were adjusted to have the same luminous flux and were edited so that the main features fit inside an oval window (3° horizontal and 4.5° vertical visual angles, respectively). The outlines of the stimuli (the edges of the faces) were not visible.

### Experiment 1 – Face recognition prior to a goal-directed saccade

**Figure 2:**
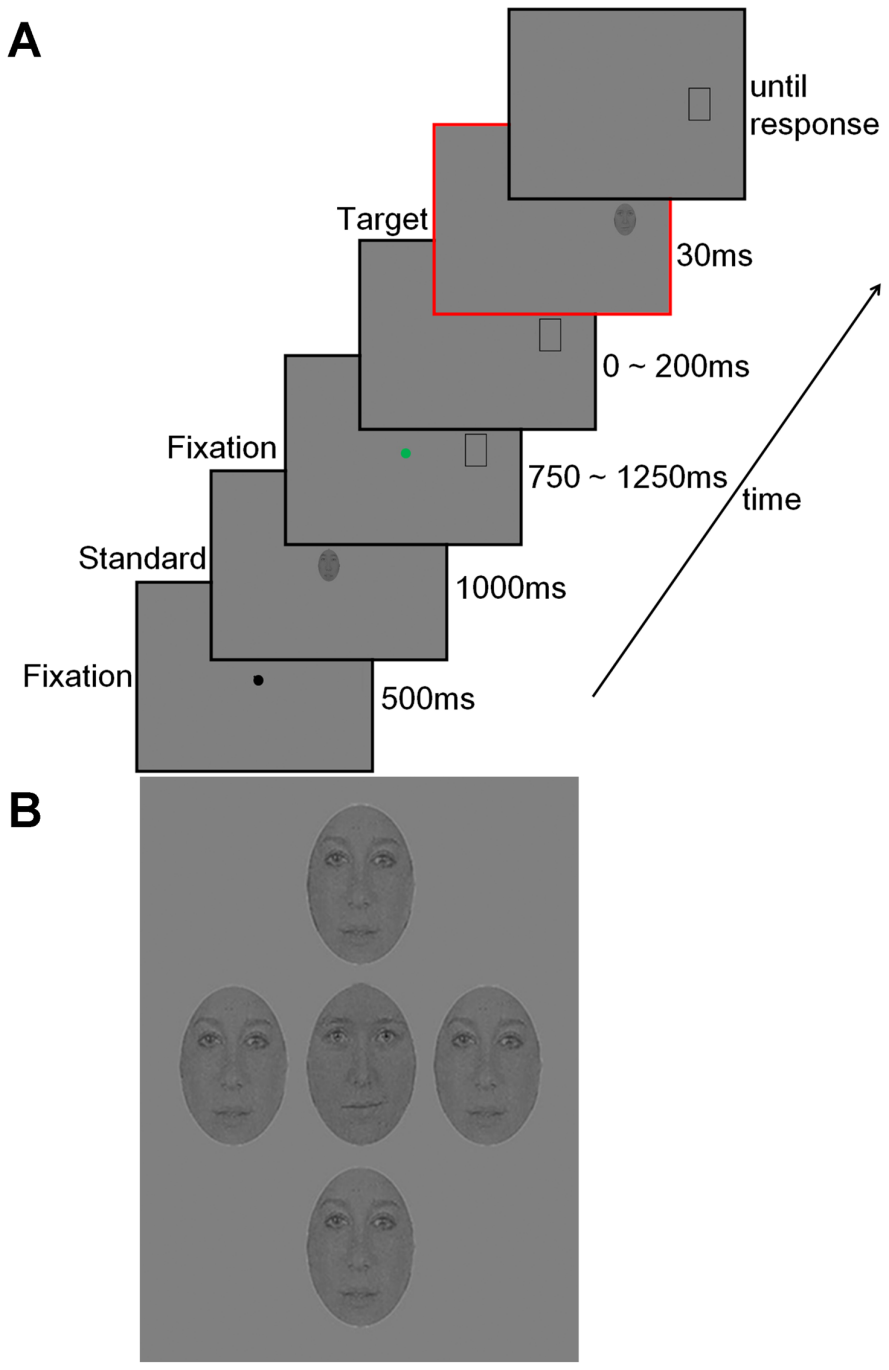
Experimental design and stimulus presentation. A) Sequence of displays in each saccade trial of Experiment 1. A placeholder (rectangles) appeared 12° to the right of the fixation spot, followed by the target face (shaded ovals). In saccade trials, the offset of the green fixation spot cued observers to make a saccade to the target face. In no-saccade trials, the fixation spot was red and observers stayed fixated on it instead. The red box outlines the target face presentation. B) The presentation of the targeted face crowded by flankers in Experiment 2. The center-to-center distance between the target and two horizontal flankers was 3.5° and the distance between the target and two vertical flankers was 5°.

Experiment 1 was designed to examine the change in face recognition before the execution of a goal-directed saccade. The experiment began with the calibration of the eye-tracker using the standard nine-point Eyelink calibration procedure. Drift corrections were performed at the beginning of each trial. After that, a fixation stimulus (black dot with diameter of 0.2°) appeared at the center of a uniform gray display. After the participants had kept fixation within a 2°×2° region centered on the fixation stimulus for 500 ms, a face, noted as the standard stimulus, was presented at the center of the screen for 1000 ms. Then, a green or red fixation spot (width  =  0.2°) replaced the face at the same center location and appeared simultaneously with the presentation of a black placeholder (3°×4.5°) 12° to the right of the fixation spot. The black placeholder indicated the location of the upcoming target face. The fixation point and placeholder were presented for a delay between 750 – 1250 ms, which was varied randomly. The colored fixation points then disappeared to cue the observer to either make a saccade to the target face (green spot, “saccade” trials) or remain fixated on the center (red spot, “no-saccade” trials). Then a pseudorandomized interval (0–200 ms) (described in detail below) was presented with only the black placeholder visible, after which a second face, noted as the target face, appeared in the position of the placeholder for a duration of 30 ms. After which the black placeholder replaced the face and remained in place while the eye tracking software monitored the saccade. The moment of the saccade was scored as time 0 and the time between the saccade, going backwards to the presentation moment of the target face was measured in milliseconds for each trial (probe time of the trial). The participants were then asked to judge whether the two faces were the same or different in a two-alternative forced-choice (2AFC) task (Fig. 2A). The participants then entered their choices manually with individual strokes on a keyboard (M for correct pairings, N for incorrect pairings).

The participants completed 360 trials (180 saccade, 180 no-saccade) in a single testing session and runs of saccade and no-saccade trials were alternated in blocks of 15. After every 60 trials, there was a break for the participants. The standard face was changed after each trial and randomly selected from the 20 images collected from the JACNeuF database. The target face was also randomly selected from the 20 images and the probability in which the two faces were the same was 0.5 for each trial. Within sessions, the target face/placeholder position was randomly shifted vertically (±1°) to prevent the participants from pre-programming eye movements (three positions with the same probability). Each observer completed two sessions in different days.

Similar to a method described previously [31], the first block in each session was always a saccade block in order to determine the time of the target presentation. And the time of the target presentation was varied in order to sample performance at different times before a saccade. The time between the fixation spot offset (the time point at which the visual stimuli were removed) and saccade onset was referred to as the saccadic latency (SL). After each saccade trial, the median saccadic latency (mSL) in the same 15-trial saccade block was calculated to be able to predict when a saccade would occur in the next trial. The mSL of the first saccade trial of the session was set to 180 ms and was used to calculate the mSL for the subsequent trials. After each saccade block, the mSL from the previous saccade block was used as the initial mSL value for the first trial of the subsequent saccade block and the mSL was recalculated. In each saccade trial, the time interval between the fixation spot offset and target onset was varied. The manipulation of the interval time between the fixation spot offset and target onset in each saccade trial was adjusted to the mSL of the present trial minus 45, 75, 105, 135, or 165 ms in a pseudorandomized manner over the present block, respectively. These same time adjustments were then used in the next corresponding no-saccade 15-trial block to allow stimulus presentation to be matched with the saccade block.

Before testing, a training session for each participant was performed which was identical to the testing sessions, except the participant was provided with auditory feedback on their performance by warning the participants in real time if they had not kept fixation within the 2°×2° region during the fixation time, if they initiated the saccade before the offset of the fixation spot, or if they didn’t make a saccade 500 ms after the offset.

### Experiment 2 – Recognition of a face in a crowd prior to a saccade

Experiment 2 was designed to examine the change in the recognition of a face in a crowd before saccadic eye movements began. All visual parameters and the task were the same as those described in Experiment 1 with the following exceptions. 1) The participants were presented with four identical faces (the flankers), different from both the standard face and the target face, simultaneously with the target. The center-to-center distance between the target and two horizontal flankers was 3.5° and the distance between the target and two vertical flankers was 5° (Fig. 2B). 2) The distance from the fixation spot to the target was different for each observer and was established by a threshold procedure prior to performing experiment 2 (9.5±0.4°). The maximum distance from fixation spot to the target to yield 75% correct performance in no flanker condition was determined by two randomly interleaved psychophysical QUEST procedures [32]. Briefly, target presentation followed a randomized delay (between 12–200 ms) after the fixation spot offset. At which point, the participants maintained a steady fixation in each trial. Detection thresholds were measured by systematically varying the target to fixation distance from trial to trial (40 trials/ QUESTs), each converging at 75% correct performance. After each experimental run, the distance output for each QUEST was plotted as a function of the trial number. Final thresholds were derived using a maximum likelihood psychometric curve fitting procedure based on the data from QUESTs [32]. All other experimental details of threshold determination trials were the same as described in Experiment 1.

### Experiment 3 – The spatial relationship of pre-saccade face recognition in a crowd

Experiment 3 was designed to examine the extent of spatial crowding of face recognition before a saccade. The differences between the methods of experiment 2 and experiment 3 were as follows. The fixation stimulus (black dot), the standard face, and the fixation spot (colored dot) were all horizontally shifted 9° to the left of the center of the screen. The target face was 10° to the right of the fixation spot. The target-flanker separations (the center-to-center distance between the target and two horizontal flankers) were manipulated over the course of the experiment. In a single session, as described in Experiment 1 above, the separation distances was held at either 3°, 3.5°, 4°, 5.5°, and 7°, respectively. The center-to-center distance between the target and two vertical flankers was adjusted accordingly and were held constant for one session. Each observer performed each session at the respective separation differences twice (one session a day) and collectively completed 3600 trials in total.

### Data preprocessing

The data from all three experiments were processed to ensure that the saccade onset matched the parameters of the experiment. The criteria for saccade onset were considered an eye movement velocity of 30°/s and an acceleration of 8000°/s^2^. The latency and endpoint of the first saccade leaving the fixation region after the fixation spot offset were determined by the DataViewer software (SR Research, Ottawa, Canada). Trials were discarded if (1) fixation was out of the 2°×2° region during the fixation time in a saccade trial or during the whole course of a no-saccade trial; (2) the latency of the first saccade was below 70 ms or above 400 ms; (3) the target appeared outside the 180 ms to 30 ms period before a saccade; (4) the target presentation was met with an eye blink; (5) the first saccade endpoint was > 3° from the target in Experiment 1 and 2, or 5° in Experiment 3. Based on these criteria, the total data used for analysis included 2712 trials (or 75.3%) in Experiment 1 and 2558 trials (or 71.1%) in Experiment 2. In Experiment 3, the data analysis included a total of 13,356 trials (or 74.2%).

### Data analysis

The group recognition performance before a saccade was evaluated for its time dependent changes by introducing an analysis of random permutation in the data according to the method described by Rolfs et al. [16], [17]. The saccade trial data was sorted into time bins according to the asynchrony of the recorded target-saccade onset times, with each bin 30 ms wide, measured backward in time from the saccade measurement time point 0 (30–59, 60–89, 90–119 ms etc). This served as the original sampling data. If the performance was truly time invariant, the performance of each time bin would not differ from that of random permutations across time. Therefore, a surrogate data set was generated by linking each of the participants’ responses to its particular target to saccade time length and randomly reassigning it to a probe time bin for each observer. This process was repeated 1,000 times to obtain a distribution of surrogate samples. Then the means and 95% confidence intervals of these surrogate samples were computed. The original data were compared with the means of the surrogate data to identify whether the average performance of the original data differed from that of the surrogate data, which indicated that the face recognition performance varied as a function of time.

For Experiment 3, the critical spacing was calculated by following the equation: pc  =  a(1 – exp(–s(d – i))), d>i, where pc is proportion correct, a is the asymptote, s is the scaling factor, d is the target-flanker separation, and i is the x-intercept [33], [34]. MATLAB (MathWorks, Natick, USA) was used to estimate all exponential parameters. The critical distance, c, was defined as the target–flanker distance at which accuracy achieved 90% of the asymptotic value, and it was calculated using the following equation: c = i – ln(0.1)/s.

## Results

### Experiment 1

**Figure 3:**
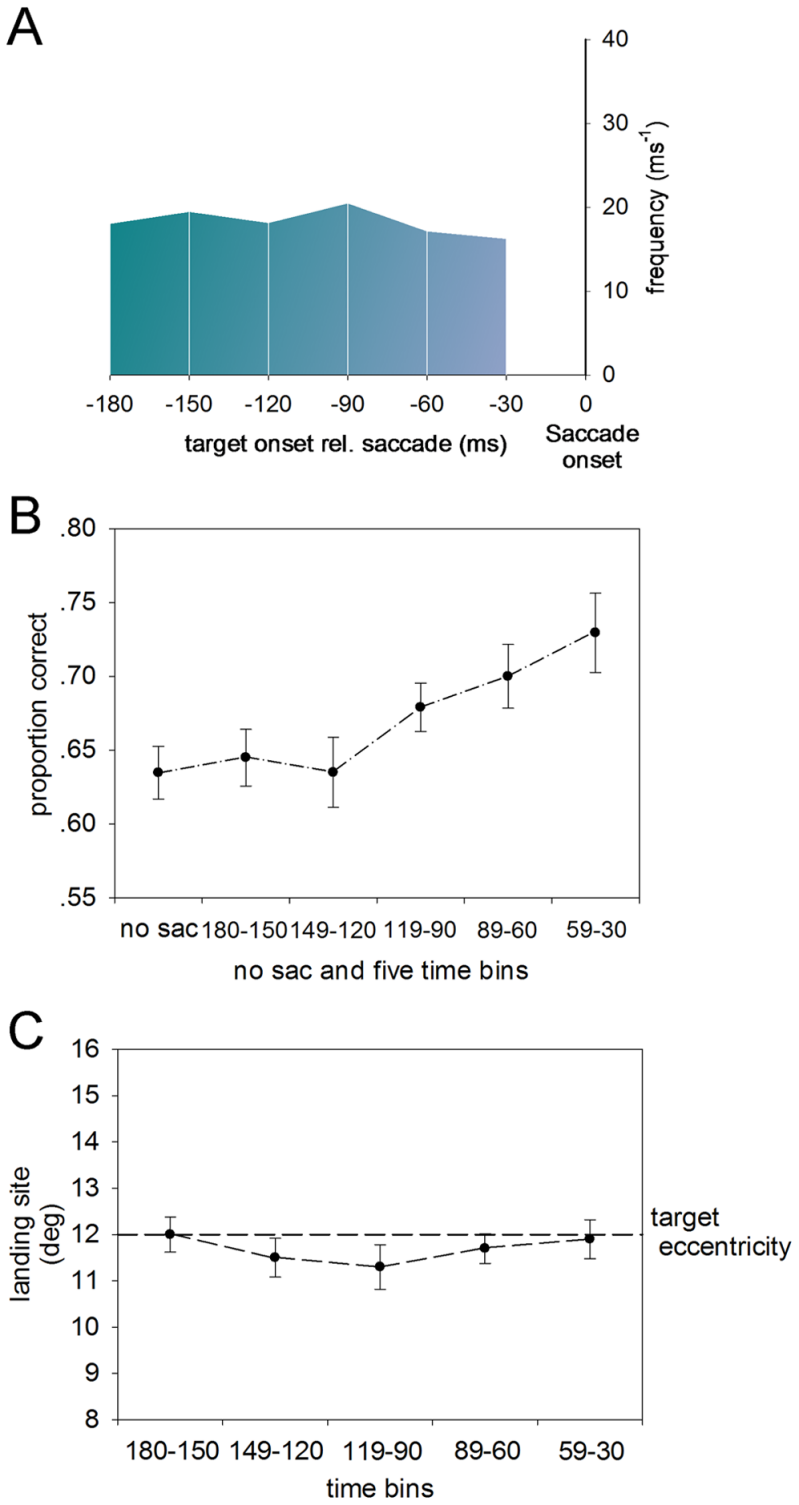
Effect of impending saccade on face perception. A) Density plot of the number of saccades in each of the five time bins. The x-axis represents the time of target presentation relative to the time of saccade onset. B) The proportion of correct judgment during the fixation period and within the five time bins before a saccade (Mean +/– S.E.M). C) Average saccade landing sites (in degree) for each of the five time bins, respectively. The dotted line stands for the distance from the center of the target to the fixation spot.

Face recognition abilities were mapped according to time frames prior to saccadic eye movements. Valid face recognition data was sorted into five separate time bins (30–59, 60–89, 90–119, 120–149, and 150–180 ms) which represented periods from the presentation of the target stimuli to the onset of the eye movement. An approximately equal number of saccade trials were spread across the time bins following data preprocessing. Figure 3A depicts a density plot of the number of saccade trials registered in each of the five time bins. In each saccade trial, there was a placeholder presented to the observer, indicating the location of the upcoming target, with which the observers were to make a directed saccade. However, only trials in which the saccade fell into the placeholder position (3°×4.5°) were analyzed. A total of 6.4%, 6.0%, 7.1%, 7.6% and 6.6% of trials were excluded from each of the five time bins, respectively.

When a face appeared in the peripheral visual field on its own in Experiment 1, the dynamics of the face perception prior to a saccade were examined and were compared to the perception of when observers maintained their fixation on the center. The discrimination accuracy for no-saccade trials was 63.5±1.8% (mean ± SEM), which was set as the baseline. As to saccade trials, the discrimination accuracies for all the five time bins were 64.5±1.9%, 63.4±2.4%, 68.1±1.6%, 70.0±2.2% and 73.2±2.7% (mean ± SEM), respectively. It was found that when the target to saccade time spacing was shortened to ∼ 120 ms before the eye movement, the face recognition performance gradually increased to be above the baseline (two-tailed paired samples t test between 119 – 90 ms time bin and the baseline, t_(4)_  = 8.57, p = 0.001; Fig. 3B). The same stimulus timing in saccade trials was used as in the no-saccade trials. However only the trials in which the target was off before the saccade began were selected in the saccade trials. This ensured that in the no-saccade and saccade trials the target presentation occurred with the eye focused on the fixation point such that the target was at the same location to the retina in both trials. Therefore the only difference between them was the approaching saccade.

Figure 3C depicts the average saccade landing sites in reference to the initial fixation spot for all the five time bins. They were 12.0±0.4°, 11.5±0.4°, 11.3±0.5°, 11.7±0.3°, 11.9±0.4° (mean ± SEM), respectively, which showed that saccades slightly underestimated the eccentricity of the target (12°). Also the average landing sites were not found to be significantly different by paired comparisons (all ps >0.1). This indicated that the locations of the saccade endpoints would not affect the interpretation of the dynamic changes of the perceptual reports.

### Experiment 2

**Figure 4:**
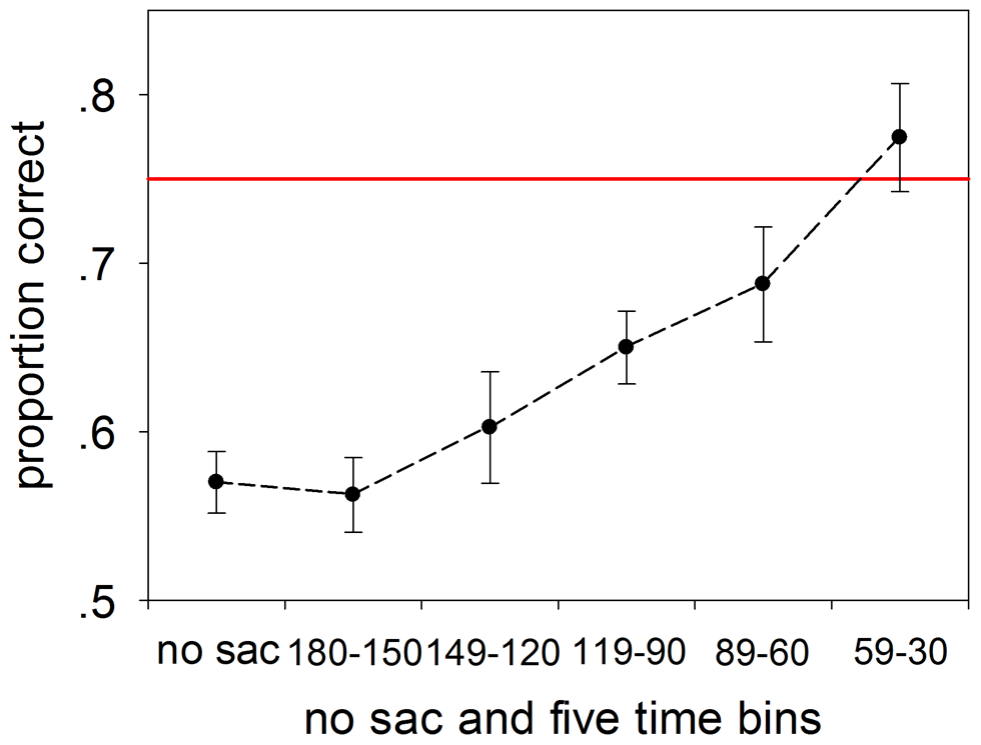
The pre-saccade recognition of a crowded face. The proportion of correct responses identifying a face flanked by four identical faces. The red line denotes 75% accuracy. All observers had met this criterion while adjusting for the eccentricity of the target by presenting the target alone without performing a planned saccade.

When a face was presented with flankers to examine the effects of identifying a crowded face in Experiment 2, the dynamics of the face perception prior to a saccade were examined and were compared to the perception of when observers maintained their fixation on the center. The eccentricity of the target for each observer was adjusted to ensure the participant’s responses to the individual face target presentations was correct in 75% of trials when no saccade was planned (see Materials and Methods). For no-saccade trials in the flanker condition, the accuracy sharply fell to 57.1±1.8% (mean ± SEM), significantly different from that of the no flanker condition (two-tailed t test between no-saccade trials in the flanker condition and trials in no flanker condition, t_(4)_ = 25.46, p<0.001), indicating that crowding was effective in impairing face recognition. While for saccade trials, similar to the results of Experiment 1, the accuracies for all the five time bins were 56.3±2.2%, 60.3±3.3%, 65.0±2.2%, 68.8±3.4% and 77.4±3.2% (mean ± SEM), respectively. It was found that when the target to saccade time spacing was shortened to ∼ 120 ms before the eye movement the recognition impairment due to the crowding of faces was gradually relieved (two-tailed paired samples t test between 119 – 90 ms time bin and no-saccade trials, t_(4)_  = 9.56, p<0.001). In particular, the accuracy of recognition of a face in a crowd in the 59 – 30 ms time bin, 77.4±3.2% (mean ± SEM), reached 75%, which was the response level under the no flanker condition in the preliminary target-fixation distance establishment trials (Figure 4). Compared to the performance in Experiment 1, there was a greater improvement of recognition just before a saccade (30–59 ms time bin): there were about 10% increment in respondent accuracy in Experiment 1 and about 20% increment in respondent accuracy in Experiment 2.

### Experiment 3

**Figure 5:**
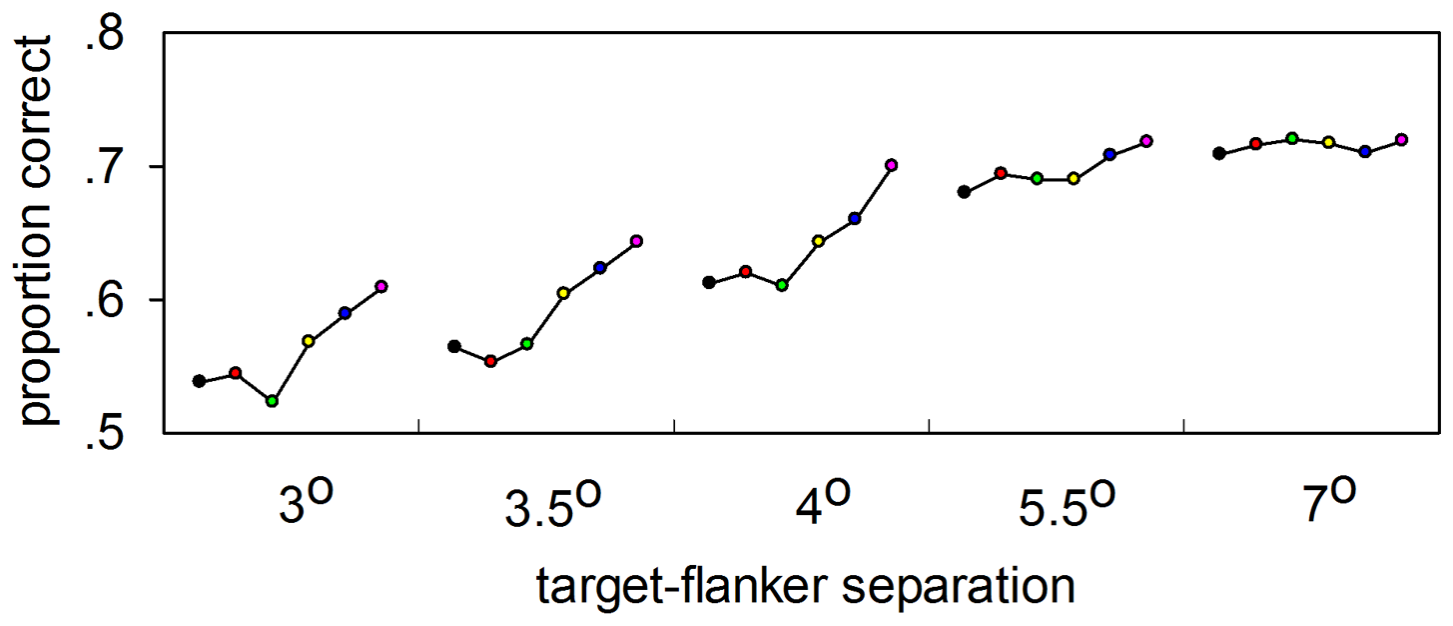
The pre-saccade recognition of a crowded face at different target-flanker separations. Differences in accuracy of pre-saccadic face recognition as a function of the time from the target presentation to the saccade onset, displayed at each target-flanker separation distance, respectively. The time bins are color coded to coordinate in figure 5: Black, no saccade; red, 180–150 ms; green, 149–120 ms; yellow, 119–90 ms; blue, 89–60 ms; pink, 59–30 ms.

Experiment 2 identified that the effect of crowding on face recognition was largely reduced just before a saccade (30–59 ms time bin). Therefore, Experiment 3 was performed to examine the effect of the extent of spatial crowding on face recognition before a saccade. Figure 5 depicts the accuracy of face recognition as a function of the time from the target presentation to the saccade onset, plotted separately for each target-flanker separation. It was found that when the flankers were closer to the target (target-flanker separations were 3°, 3.5° or 4°), from ∼ 120 ms before the first saccade the accuracy of responses gradually improved and peaked in the 59 – 30 ms time bin. However, when the distance between the target and flankers was larger (target-flanker separations were 5.5° or 7°), the accuracy did not change across the various time bins (by the analysis of random permutation, see Materials and Methods). But the proportions of correct responses were higher than those with smaller degrees of separation.

**Figure 6:**
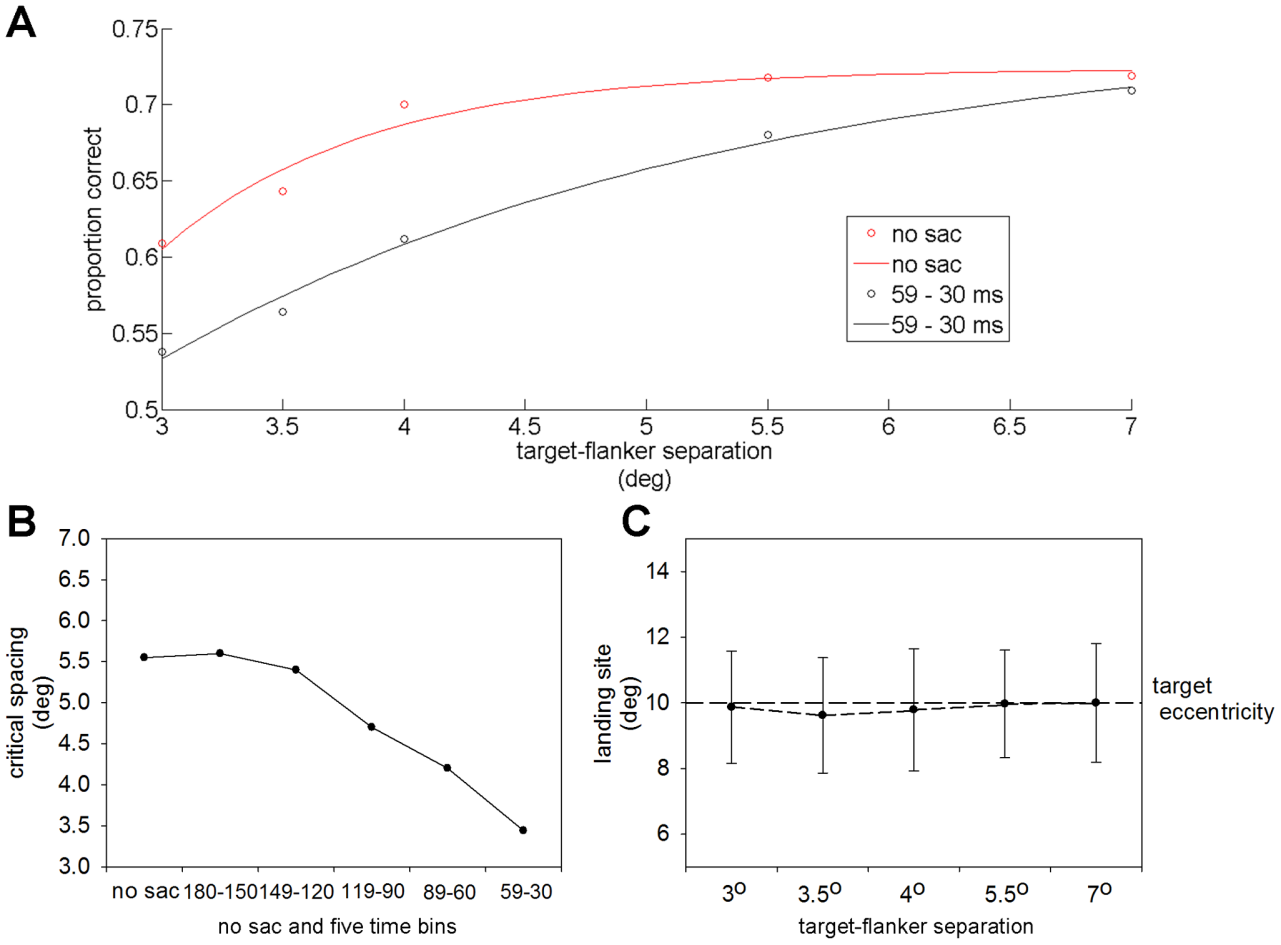
The analysis of the result in Experiment 3. A) Two simulated exponential curves of the accuracy rate at each target-flanker separation distance: 59 – 30 ms time bin data (red curve); no-saccade condition data (black curve). B) The temporal evolution of critical spacing prior to a saccade. C) Average saccade landing sites for different target-flanker separations, respectively. Error bars stand for 1.95*SEM, which indicate a range covering 95% of the landing sites.

Based on the data in Figure 5, an exponential curve was used to simulate the data and the critical spacing was calculated (see Materials and Methods). The simulated curves for the 59 – 30 ms time bin and for the no-saccade condition are plotted as examples in Figure 6A. The improvement in face recognition during the saccadic preparation is identified by the upward shift in the proportion of correct responses (red curve compared to black curve). Figure 6B depicts the temporal evolution of the critical spacing calculated from the simulated curves. The critical spacing for the no-saccade trials was 5.4°, which corresponded to a proportion of 0.54 (critical spacing/eccentricity). This value was in agreement with Bouma’s law [20], and was similar to values of the critical spacing found by other groups studying on shapes, orientation and faces [25], [35]. In addition, the critical spacing drastically decreased with the change in target-saccade onset time measured prior to a saccade and reached the minimum 3.4° in the 59 – 30 ms time bin. This decrease started ∼ 120 ms before the saccade.

To rule out the possibility that the differences in saccade landing sites between each target-flanker separation contributed to the different visual performance for each target-flanker separation, the average landing site and the range covering 95% of landing sites for each target-flanker separation were calculated (Fig. 6C). Saccade endpoints were found to fall almost the same distance from the target center, revealing that oculomotor accuracy was unaffected by different target-flanker separations. Furthermore, the length of the landing range was ∼ 3.5°, which was similar to the minimum of the critical spacing in the 59 – 30 ms time bin. This indicated that the oculomotor accuracy was strongly coupled to the extent of spatial crowding.

## Discussion

It was observed in this study that rapid pre-saccadic increases in the recognition of faces occurred as the saccadic movement approaches, whether or not they were crowded with other faces. However, the extent of spatial crowding between target faces and flankers had a dramatic effect on the recognition just before the eye movement. Interestingly, the changes in face recognition capabilities and the critical spacing had similar temporal dynamics: starting ∼ 120 ms before a saccade. At this point, the participants were then better able to identify the faces as the target presentation got closer to the eventual saccade onset. It is thought that just before the eyes move, observers perceive a higher contrast of an object’s orientation at the saccade’s target location to the background and become more sensitive to the orientation differences [17]. However, faces, as the most important and salient visual stimulus a human encounters, are a special kind of object, which are thought to be processed in a holistic manner [11]. Our results indicate that increases in high-level complex perception, in addition to low-level elementary features, occur immediately before saccade onset at multiple levels in the visual system. This increased perception is, thus, an integrative process for the brain’s visual network.

With regard to complex objects, such as faces, this perception is related to the separation of individual parts from the whole. This is expanded to the objects’ surrounding environment, where elementary or low level components (such as orientation or color) are interwoven in the visual field with complex elements or objects. Often, the perception of the object and environment is thought to be ordered in a hierarchical fashion. However, it is not clear whether these groupings are processed by the brain as parallel or successive stimuli. The focus of previous research has viewed the processing of the whole and low level components to occur through different neural networks or pathways. In the past, this processing was even considered to occur in different cerebral regions [36], [37]. This suggests, and recent evidence supports the notion, that the brain relies on distributed processing pathways for complex items and their parts [38], [39]. Interestingly, the results of our experiments and others recently performed on standard objects and orientations investigating recognition during saccade preparation, show that the processing of orientations and faces increases at the same time before the saccade. This suggests that low and high levels of information are processed in a similar way during saccade preparation.

A face seldom appears alone in the natural environment. Instead, they often come together in a cluster and humans need to correctly identify a target face with accurate eye movements. In this study, temporal dynamics of the critical spacing of faces surrounded by other faces were observed, which indicated a change in the extent of spatial crowding. This suggests that prior to the saccade the eye may be adjusting the overall crowd background to focus on one point in space. Imagine that there is a group of people standing far on your right hand side while your eyes are focused on other places; just then a new person joins the group in your peripheral view, such that your gaze will turn to look at the new person. At the very beginning, you only know the “group” because of all the crowded faces, but just before the eye movement, preprocessing of the crowd may relieve the information load to help you recognize that person quickly.

These findings, and the anecdote, bring to light an interesting question into the possible neural basis for this pre-saccadic processing. Previous neurophysiological studies have shown that prior to an eye movement the receptive fields of brain area V4 move towards the eventual saccade target location and the field contracts [40]. The V4 brain area is regarded as an important crowd information processing region [41], [42] and the ventral V4 as related to the processing of faces [43], [44]. Moreover, responses to visual stimuli in brain area V4 have been shown to improve after short electrical stimulations at currents that are below the saccade evoking threshold. These stimulations occurred within the frontal eye field (FEF) which plays an important role in the control of voluntary eye saccades [45]–[48]. The magnitude of the response improvement in these previous studies was related to both the importance of the stimuli in the receptive field and the competing stimuli present outside the field. This phenomenon may explain the greater rates of face recognition in Experiment 2 than in Experiment 1 as the crowd information may provide this competing stimuli.

The fact that observers have perceptual changes in the moments prior to a saccade reveals a tight interaction between sensation and movement. It is likely that the increased accuracy of the perception of low-level elementary features [49] and the complex representations of faces at the immediate landing site just prior to the eye movement may aid the targeting of the eye as well as facilitate the coming visual processing at the fovea. It is possible that this phenomenon acts to stabilize the pre-saccadic visual representation with the post-saccadic one as the saccade occurs. All told, this investigation highlighted an interesting aspect and provided a new realization about early face recognition mechanisms. However, is this pre-saccadic processing unique to faces, what about other complex objects? And, what are the relations between the perception of low-level elementary features and that of high-level complex representations of faces during saccade preparation, with their similarities in time course of changes? To answer these questions, much work still lies ahead.
